# Supplementary material for: Myriad Mapping of nanoscale minerals reveals calcium carbonate hemihydrate in forming nacre and coral biominerals
Source: Nat Commun. 2024 Feb 28;15:1812. doi: 10.1038/s41467-024-46117-x (PMC10901822; doi:10.1038/s41467-024-46117-x)
Supplement: Supplementary file 1 — Supplementary Information [file 41467_2024_46117_MOESM1_ESM.pdf]

Supplementary Information
for
Myriad Mapping of nanoscale minerals reveals calcium carbonate hemihydrate in forming nacre and coral biominerals

Connor A. Schmidt[1], Eric Tambutté[2], Alexander A. Venn[2], Zhaoyong Zou[3], Cristina Castillo Alvarez[4], Laurent S. Devriendt[4], Hans A. Bechtel[5], Cayla A. Stifler[1], Samantha Anglemyer[1], Carolyn P. Breit[1], Connor L. Foust[1], Andrii Hopanchuk[1], Connor N. Klaus[1], Isaac J. Kohler[1], Isabelle M. LeCloux[1], Jaiden Mezera[1], Madeline R. Patton[1], Annie Purisch[1], Virginia Quach[1], Jaden S. Sengkhammee[1], Tarak Sristy[1], Shreya Vattem[1], Evan J. Walch[1], Marie Albéric[6], Yael Politi[7], Peter Fratzl[8], Sylvie Tambutté[2], Pupa U.P.A. Gilbert[1,4,9#*]

Table of Contents

Supplementary Tables **2**
Supplementary Table 1 2
Supplementary Table 2 3
Supplementary Table 3 4
Supplementary Table 4 6
Supplementary Table 5 8
Supplementary Table 6 9

Supplementary Figures **10**
Supplementary Fig. 1. 10
Supplementary Fig. 2. 11
Supplementary Fig. 3. 13
Supplementary Fig. 4. 14
Supplementary Fig. 5. 15
Supplementary Fig. 6. 16
Supplementary Fig. 7. 17
Supplementary Fig. 8. 18
Supplementary Fig. 9. 19
Supplementary Fig. 10. 20
Supplementary Fig. 11. 21
Supplementary Fig. 12. 22

Supplementary Files **23**

Supplementary References **23**

Supplementary Tables

Supplementary Table 1

mineral phase in Cni16 component spectra	color in spectra and MMs	origin	# acquisitions	# single-pixel spectra
0-ACCH_2O	R	synthetic	2	3200
1-ACC	G	synthetic	2	3200
2-CCHH	C	synthetic	2	20402
3-MHC	M	synthetic	1	2254
4-Vaterite	Y	synthetic	4	4409
5-Aragonite	B	synthetic	4	4056
6-Calcite	B	synthetic	4	3707

Supplementary Table 1. List of Cni16 x-ray absorption spectra, the colors with which these mineral phases are identified in spectra and Myriad Maps (MMs) of mesoscale carbonate-phases, their origin, the number of acquisitions from which single-pixel spectra were extracted, and the number of single-pixel spectra that were averaged and peak-fitted to obtain each component spectrum. Carbonate MMs from freshly deposited biominerals are displayed in Figs. 2, 3, Supplementary Figs. 4-6, 10-11, and use the same color scheme of this table. Aragonite and calcite are frequently omitted for clarity. Note that the color scheme in Supplementary Fig. 12 is slightly different than that of the MMs in order to present mixed phases, as displayed by the color legend.

Supplementary Table 2

	synthetic aragonite	bulk coral skeleton	bulk nacre	synthetic calcite	sea urchin spine
average χ^2 obtained with 5cmp biogenic (Cni14) component spectra	0.0035	0.0054	0.0038	0.0024	0.0042
average χ^2 obtained with 5cmp synthetic (Cni16) component spectra	0.0027	0.0046	0.0034	0.0024	0.0044
T Test comparing biogenic (Cni14) and synthetic (Cni16), p of null hypothesis	2.9×10^{-3}	9.8×10^{-3}	2.7×10^{-2}	0.45	0.11

Supplementary Table 2. Average χ^2 values from a 6-pixel line analyzed in each sample (specified in each column head) using the best analysis resulting from Supplementary Table 3, which is 5cmp analysis, using either biogenic component spectra (Cni14) or synthetic component spectra (Cni16), and T Test results, obtained using 2-tailed, two-sample unequal variance, describing the probability of the null hypothesis that the two compared results are not significantly different ($p > 0.05$). The fit improves significantly ($p < 0.05$) for all aragonite minerals when using the synthetic spectra Cni16, which is important because CCHH is observed mostly in aragonite biominerals. In synthetic calcite, instead, fitting with biogenic or synthetic spectra does not make a significant difference ($p = 0.45$). In sea urchin spines using synthetic rather than biogenic spectra worsens the fit slightly (χ^2 goes from 0.0042 to 0.0044, $p = 0.11$). Due to the low frequency of the new metastable crystalline phases in sea urchin spines, this decrease did not affect the results or their interpretation. With both sets of spectra CCHH and MHC are found only in trace amounts in sea urchin spines.

Supplementary Table 3

# acquisitions	tested crystalline precursor	#pixels with >90% of the tested crystalline precursor in 6cmp analysis with $ACCH_2O$, ACC, CCHH, MHC, vaterite, and aragonite or calcite	Average reduced χ^2 with 3cmp in crystalline precursor pixels<br><br>0-$ACCH_2O$<br>1-ACC<br>5-arag or 6-calc	Average reduced χ^2 with 4cmp with CCHH in crystalline precursor pixels<br><br>0-$ACCH_2O$<br>1-ACC<br>2-CCHH<br>5-arag or 6-calc	Average reduced χ^2 with 4cmp with MHC in crystalline precursor pixels<br><br>0-$ACCH_2O$<br>1-ACC<br>3-MHC<br>5-arag or 6-calc	Average reduced χ^2 with 4cmp with vaterite in crystalline precursor pixels<br><br>0-$ACCH_2O$<br>1-ACC<br>4-vaterite<br>5-arag or 6-calc	**Average reduced χ^2 with 5cmp with CCHH and MHC in crystalline precursor pixels**<br><br>**0-$ACCH_2O$**<br>**1-ACC**<br>**2-CCHH**<br>**3-MHC**<br>**5-arag or 6-calc**	Average reduced χ^2 with 6cmp with CCHH and MHC in crystalline precursor pixels<br><br>0-$ACCH_2O$<br>1-ACC<br>2-CCHH<br>3-MHC<br>4-vaterite<br>5-arag or 6-calc
8	2-CCHH	1740	0.0083	0.0069	0.0074	0.0084	**0.0069**	0.0069
8	3-MHC	194	0.0104	0.0091	0.0083	0.0105	**0.0083**	0.0084
8	4-vaterite	295	0.0072	0.0069	0.0072	0.0069	**0.0069**	0.0068
				Z Test comparing 4cmp with CCHH to 3cmp: p of null hypothesis	Z Test comparing 4cmp with MHC to 3cmp: p of null hypothesis	Z Test comparing 4cmp with vaterite to 3cmp: p of null hypothesis	**Z Test comparing 5cmp with CCHH and MHC to 3cmp: p of null hypothesis**	Z Test comparing 6cmp with all crystalline precursors to 5cmp with CCHH and MHC only: p of null hypothesis
	2-CCHH			$8.2x10^{-213}$	$2.2x10^{-95}$	0.03	**$3.0x10^{-207}$**	0.69
	3-MHC			$6.9x10^{-24}$	$4.1x10^{-80}$	0.34	**$8.0x10^{-86}$**	0.45
	4-vaterite			$3.9x10^{-3}$	1.0	0.02	**$7.0x10^{-3}$**	0.63

Supplementary Table 3. Comparison of average reduced χ^2 values obtained by fitting spectra from 10% of all areas analyzed. The 10% includes: 4 areas from coral, 2 from nacre, 2 from sea urchin spines. The subset of spectra used in the averages were chosen based on their assignment in 6 component analysis (6cmp) using the amorphous phases, the mature biomineral phase, and all 3 potential crystalline precursors (CCHH, MHC, and vaterite). The reduced χ^2 takes into account the different numbers of components used across

different analyses, so the increased degrees of freedom introduced by more components is naturally weighted to be less favorable (see methods, Equation 1). The average reduced χ^2 values were compared using the p-value of a two-tailed Z Test to determine if they were significantly different from one another, that is, if the fit improved or not by introducing the additional component spectra, compared to previous studies that only used amorphous component spectra (3cmp). The Z Test shows the probability of the null hypothesis, that is, that the fit with new components CCHH, MHC, or vaterite does *not* improve the fit. The smaller this probability the more significant the difference between the two groups compared. A probability $p < 0.05$ is considered significant. This probability is negligibly small for 4cmp analyses using CCHH or MHC ($p = 8.2 \times 10^{-213}$ and $p = 6.9 \times 10^{-24}$, respectively) in pixels assigned to either CCHH or MHC compared to 3cmp with amorphous precursors only. Thus, the great improvements of adding CCHH or MHC to the fit are statistically extremely significant. Conversely, 4cmp with vaterite the fit is slightly ($p = 0.03$) different for CCHH (in fact the reduced χ^2 is slight worse) and not significantly different for MHC ($p = 0.34$) compared to 3cmp analysis, meaning that the vaterite spectrum does not effectively describe the unknown pixels and is only identified in pixels that could be better described by 3cmp. This is also shown in Fig. 1. We then tested 5cmp analysis, introducing both CCHH and MHC, and found this to be a significant improvement compared to 3cmp with amorphous precursors only ($p = 3.0 \times 10^{-207}$ and $p = 8.0 \times 10^{-86}$). The addition of vaterite, resulting in 6cmp analysis does not provide a significant improvement in CCHH or MHC pixels ($p = 0.69$, $p = 0.45$), not even in vaterite pixels ($p = 0.63$). Thus, we excluded vaterite from all analysis in this work. The 5cmp analysis gave by far the best results, always yielding the smaller reduced χ^2 values compared to 4cmp analyses, thus, this is the analysis done for all data presented here. Separate data for the 2,229 pixels analyzed in this table and their statistical analysis are provided in file Supplementary Data 2.xlsx.

Supplementary Table 4

fit Cni16 spectra with	fit parameter	0-ACCH_2O	1-ACC	2-CCHH	3-MHC	4-Vaterite	5-Aragonite	6-Calcite
2nd order polynomial background	p0	-0.222	-0.222	-0.222	-0.222	-0.222	-0.222	-0.222
	p1	-0.017	-0.017	-0.017	-0.017	-0.017	-0.017	-0.017
	p2	0.002	0.002	0.002	0.002	0.002	0.002	0.002
peak 1 Lorentzian	amplitude	10.730	9.156	10.123	10.706	8.330	7.007	7.115
	position	352.553	352.576	352.584	352.600	352.594	352.581	352.604
	width	0.736	0.624	0.724	0.747	0.551	0.463	0.486
peak 2 Lorentzian	amplitude	1.282	2.038	2.595	3.685	1.938	0.430	2.209
	position	351.502	351.400	351.494	351.580	351.461	351.720	351.388
	width	0.722	0.750	0.835	0.992	0.572	0.430	0.421
peak 2’ Lorentzian	amplitude						0.740	
	position						351.320	
	width						0.530	
peak 3 Lorentzian	amplitude	7.918	6.762	6.957	6.872	5.868	5.085	5.315
	position	349.253	349.246	349.243	349.245	349.242	349.264	349.271
	width	0.623	0.514	0.569	0.593	0.459	0.402	0.464
peak 4 Lorentzian	amplitude	1.257	1.326	1.800	3.054	1.021	0.400	1.594
	position	348.090	348.363	348.244	348.277	348.132	348.450	348.018
	width	1.153	0.973	0.951	1.200	0.562	0.400	0.606
peak 4’ Lorentzian	amplitude			0.300		0.172	0.440	0.107
	position			347.571		347.026	347.730	347.350
	width			0.592		0.486	0.600	0.425
peak 4’’ Lorentzian	amplitude			0.300		0.071	0.200	0.196
	position			347.091		347.026	347.100	346.900
	width			0.477		0.264	0.500	0.500
peak 4’’’ Lorentzian	amplitude			0.210				
	position			346.107				
	width			0.502				
arctangent 2 (L_2 edge)	amplitude	0.800	0.800	0.800	0.800	0.800	0.800	0.800
	position	352.350	352.350	352.350	352.350	352.350	352.350	352.350
	width	0.200	0.200	0.200	0.200	0.200	0.200	0.200
arctangent 3 (L_3 edge)	amplitude	0.300	0.300	0.300	0.300	0.300	0.300	0.300
	position	349.000	349.000	349.000	349.000	349.000	349.000	349.000
	width	0.200	0.200	0.200	0.200	0.200	0.200	0.200

Supplementary Table 4. Fit coefficients for the peak fitted Cni16 component spectra presented in Figs. 1, 2, Supplementary Fig. 1. The background is a second-order polynomial with p0, p1, p2 coefficients. All fit parameters were set free, except for those shaded in gray, which were held fixed during peak fitting. Peak amplitude is the area under the Lorentzian curve; thus, amplitudes are different for different minerals, even for peak 1, with intensity 10 for all minerals. Aragonite and ACCH$_2$O have the narrowest and broadest peak 1, respectively, thus, their amplitudes are the most different. Peak position, expressed in eV, is the binding energy of the electron or the photon energy position of each Lorentzian or arctangent center; the peak or arctangent width is also expressed in eV. Note that the peak positions listed here are for the individual peaks alone, thus the positions of the peaks in the complete spectrum, with all peaks partly overlapping may appear to move a little compared to the positions listed here. Peaks are labeled as in Figs. 1, 2, Supplementary Fig. 1.

Supplementary Table 5

A	unmasked volume in first acquisitions (μm^3)	volume of precursors ($ACCH_2O$, ACC, CCHH, or MHC) in first acquisitions (μm^3)	%precursor volume for $ACCH_2O$	%precursor volume for ACC	%precursor volume for CCHH	%precursor volume for MHC	%precursor volume that transitions thermodynamically downhill (Fig. 4) or remains identical, comparing 1st and 2nd acquisitions
coral	257	0.17	5%	39%	46%	9%	89%
nacre	11	0.16	5%	36%	48%	12%	83%
sea urchin spines	43	0.06	4%	90%	3%	2%	96%
total	311	0.39	5%	45%	41%	10%	87%

B

Transition	from $ACCH_2O$	from ACC	from CCHH	from MHC
to $ACCH_2O$	1%	1%	1%	0.4%
to ACC	1%	13%	4%	1%
to CCHH	0.4%	2%	16%	2%
to MHC	0.3%	1%	2%	3%
to aragonite/calcite	3%	29%	18%	3%

Supplementary Table 5. A. occurrence of each precursor phase across all areas in each biomineral, broken down by percent of all precursor volume. The volume was calculated by multiplying the number of pixels by the known pixel size by the probing depth of 3 nm[1]. Note that the total unmasked volume, 311 μm^3, includes ~23% of the total 1.95 x 10^8 pixels analyzed, meaning that on average ~23% of each field of view was occupied by a biomineral. The most abundant precursor phase in coral and nacre was CCHH, followed by ACC, then MHC, then $ACCH_2O$, whereas in sea urchin spines the most abundant precursor phase was ACC, then $ACCH_2O$, then CCHH, then MHC. Note the significantly larger percentage of CCHH in the coral skeletons and nacre tablets compared to sea urchin spines, indicating that CCHH is a much more common precursor to aragonite than calcite. B. The percentage of precursor pixels that stay the same are highlighted in the color used in all figures for that phase. Percent pixels that undergo all other transitions in repeat movies, from each phase to all other phases, are indicated in the table in non-shaded cells. The pixels that transition thermodynamically uphill/downhill are above/below the colored diagonal of no transitions. The great majority of MM pixels go thermodynamically downhill, as also shown in the last column in A.

Supplementary Table 6

mineral phase XRD	sample ID	XRD date yyyymmdd	initial solution Na_2CO_3 mM	$CaCl_2$ mM	$MgCl_2$ mM	precipitation temperature °C	sample heating temperature °C	crystallization temperature °C	water content $CaCO_3.xH_2O$
$ACCH_2O$	LSD_009a	20230511	25	25	-	25	-	330	0.76
ACC	LSD_009a_h135	20230227	25	25	-	25	200	330	0.15
CCHH	ZZ CCHH	20230511	40	32	8	25	-	-	-
MHC	LSD_015	20230511	50	40	10	25	-	-	-
aragonite	LSD_002	20230511	25	25	-	80	-	-	-
calcite	LSD_001f_h2o	20221118	25	25	-	25	-	-	-
aragonite	CAS19	20210608	-	10	50	25	-	-	-
calcite&vaterite	CAS43	20220308	-	5	-	25	-	-	-

Supplementary Table 6. Mineralogical and chemical characteristics of the synthetic $CaCO_3$ samples for SINS-FTIR analysis above, and PEEM analysis below. Water contents and crystallization temperatures were measured by TGA/DSC.

Supplementary Figures

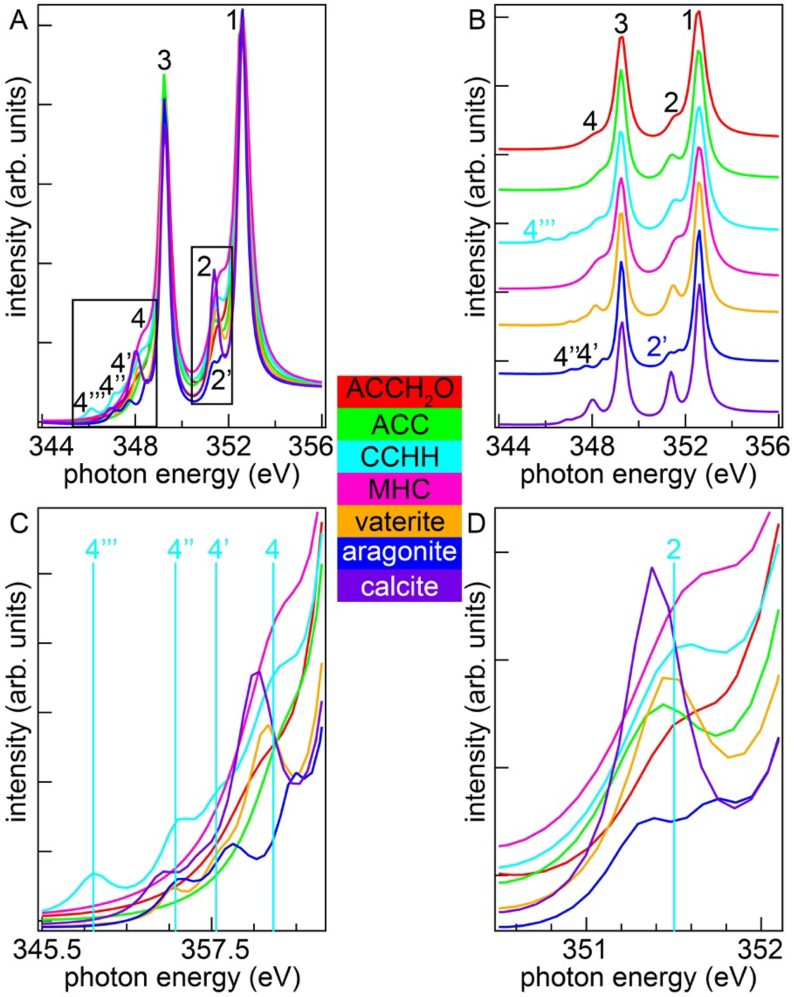

Supplementary Fig. 1. Calcium carbonate minerals have distinct spectra at the Ca L-edge. These are the Cni16 spectra used to obtain all mesoscale maps in this work, including: $ACCH_2O$, ACC, CCHH, and MHC displayed in red, green, cyan, and magenta, here and in all mesoscale maps (Figs. 2-3, Supplementary Figs. 4-6, 10-11). Aragonite and calcite are displayed in blue and purple here only, and for clarity they are not displayed in Figs. 2B, 3, Supplementary Fig. 10. Aragonite or calcite are displayed in Fig. 1A, and Supplementary Figs. 4-6, where they are both displayed in blue. We additionally show the spectrum for vaterite in yellow, used during analysis of the χ^2 (Supplementary Table 3), but eventually removed from the analysis for all other data presented here because it never improved the χ^2. The spectra are overlapped to show spectroscopic lineshape differences in A, and displaced vertically in B, for clarity. Peaks 4’ and 4’’ are labeled on the aragonite spectrum where they are most prominent but are present in all crystalline spectra except MHC (B). Peak 2 is the most distinct in all carbonates, as shown in D, where spectral regions boxed in A are magnified. Peak 2 for CCHH occurs at 351.5 eV, distinct from all other carbonates except for $ACCH_2O$. The latter is very different from CCHH, in the peak 4 region: $ACCH_2O$ has only a broad and low shoulder at 348.09 eV, CCHH instead has four distinct peaks, termed 4, 4’, 4’’, 4’’’ at very different anergy positions and with much smaller peak width. The amplitude of peak 4 at 348.24 is much greater for CCHH than for $ACCH_2O$, and the amplitude for 4’, 4’’, 4’’’ are much smaller than peak 4 for $ACCH_2O$. All peak parameters are listed in Supplementary Table 4. Importantly, all Cni16 spectra had precisely the same background polynomial, and the same arctangent absorption thresholds, to eliminate unphysical fluctuations in mesoscale myriad carbonate phase mapping. Panels C and D magnified regions of peaks 4 and 2, respectively. Peaks labels here are identical to those in Supplementary Table 4. Peaks that only exist in certain spectra (e.g., peak 4’’’ in synthetic CCHH or 2’ in aragonite) are labeled near the relevant peak and spectrum in panels B,C,D, and they are displayed in the same color as the spectrum in which they appear. The Cni16 spectra are provided in file Supplementary Data 1.xlsx.

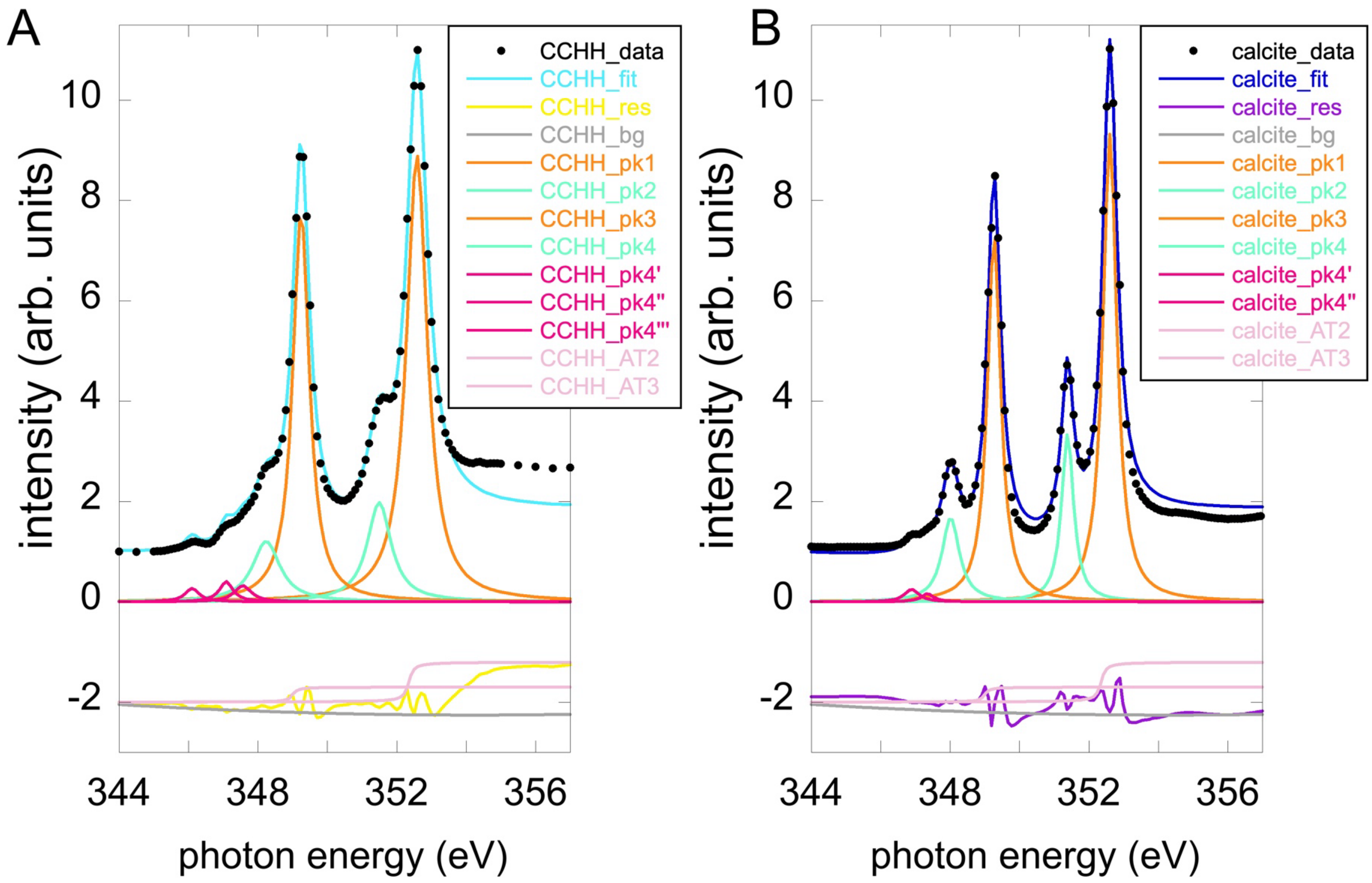

Supplementary Fig. 2. Peak-fitted Ca L-edge spectra for CCHH and calcite. Peak fitting results for A. CCHH and B. calcite, broken down by each Lorentzian peak, arctangent, and polynomial, as presented numerically in Supplementary Table 4. Each individual element adds up to produce the fit, which is used as the component for myriad mapping. A. shows the slight exaggeration of peaks 4, 4’, 4’’, and 4’’’ in the fit relative to the data. Discrepancies between data and fit in the post-edge come from the normalization of the

peak 1 intensity to 10 in all spectra while using the same arctangents and polynomials, which leads to fits based on lineshape rather than concentration of Ca.

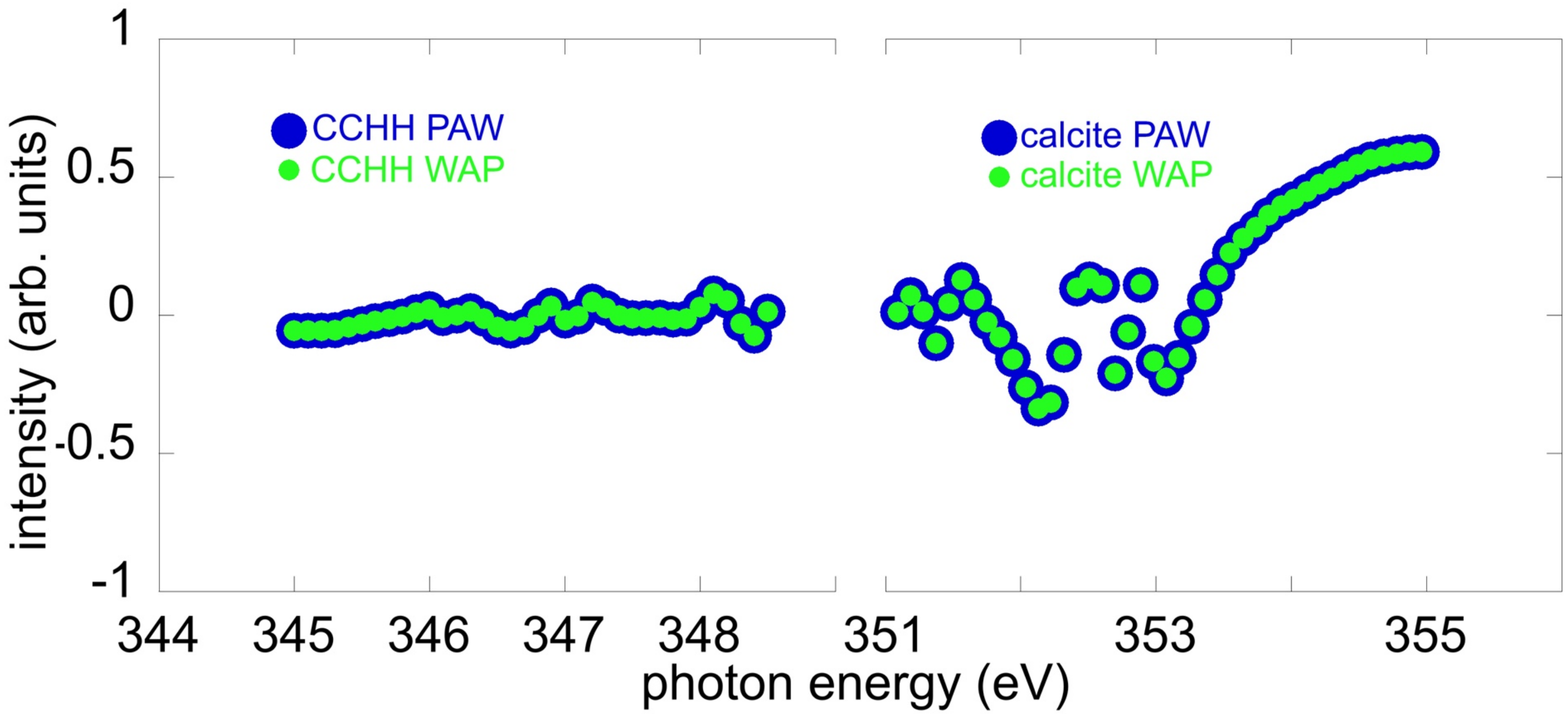

Supplementary Fig. 3. Details of the peak-fitting procedure. Residue between data and fit for calcite peak 2 and CCHH peaks 4-4''' either fitting position (P) first, then amplitude (A), then width (W), (PAW, displayed in blue) or in reverse order (WAP, displayed in green). There is almost perfect overlap of the two, showing that the order in which parameters are freed to fit does not affect the final fit.

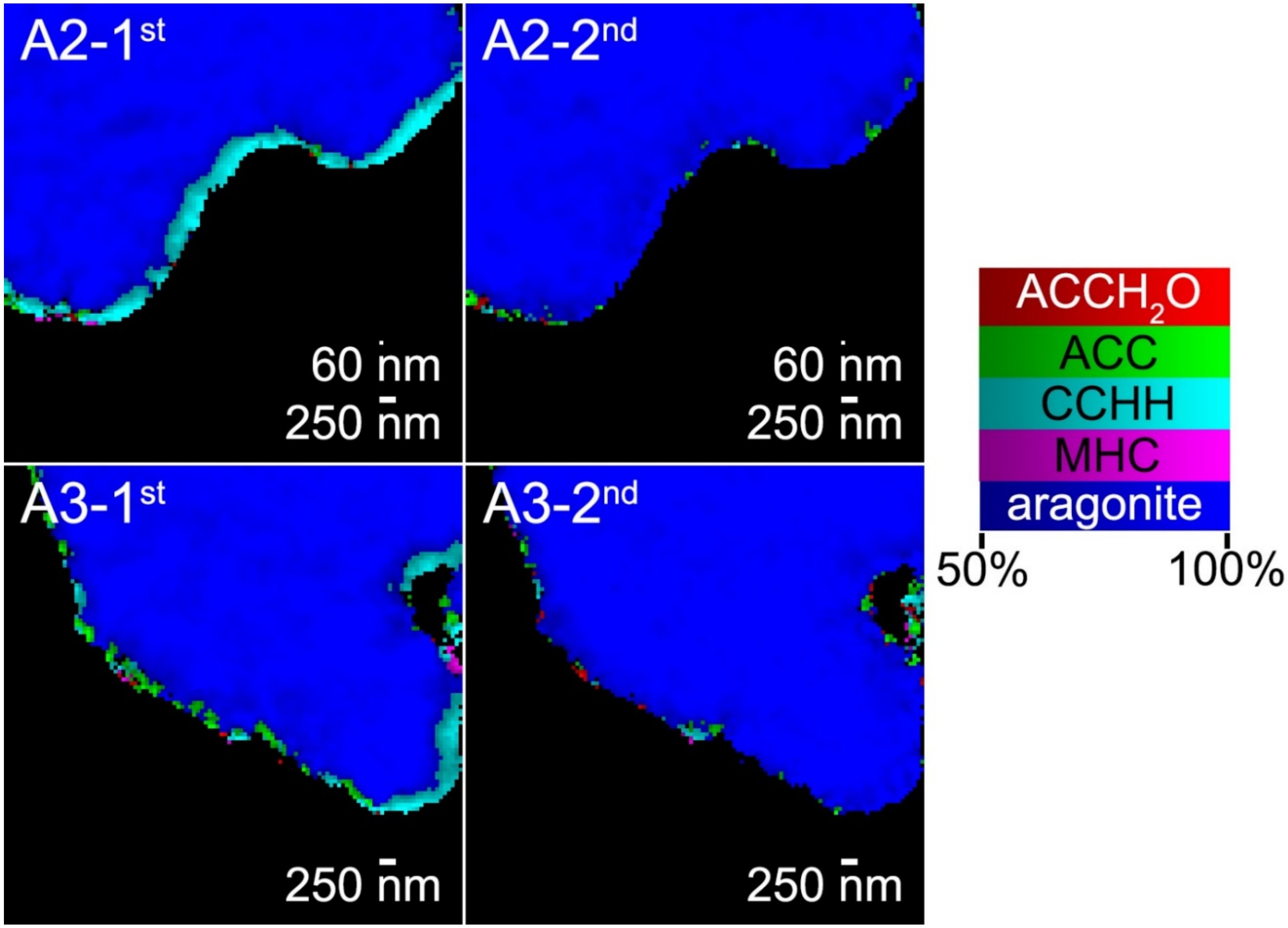

Supplementary Fig. 4. Repeat acquisitions of the same coral skeleton area show phases transforming thermo-dynamically downhill. Myriad Mapping (MM) of mesoscale carbonate-phases for the same area of coral skeleton shown in insets A2 and A3 in Fig. 3. The first Ca acquisition is shown on the left and labeled A2-1st, the repeat is A2-2nd. Similarly, for A3-1st, and A3-2nd acquisitions. All mesoscale maps were obtained using the 5 components indicated in the color legend, and only pixels containing greater than 50% of each component are displayed in the corresponding color. Note that most ACC and CCHH pixels in 1st acquisitions transformed into aragonite in "2nd" panels. The single pixel scalebar is excluded from panels A3-1st and A3-2nd to avoid covering precursor pixels but is the same pixel size.

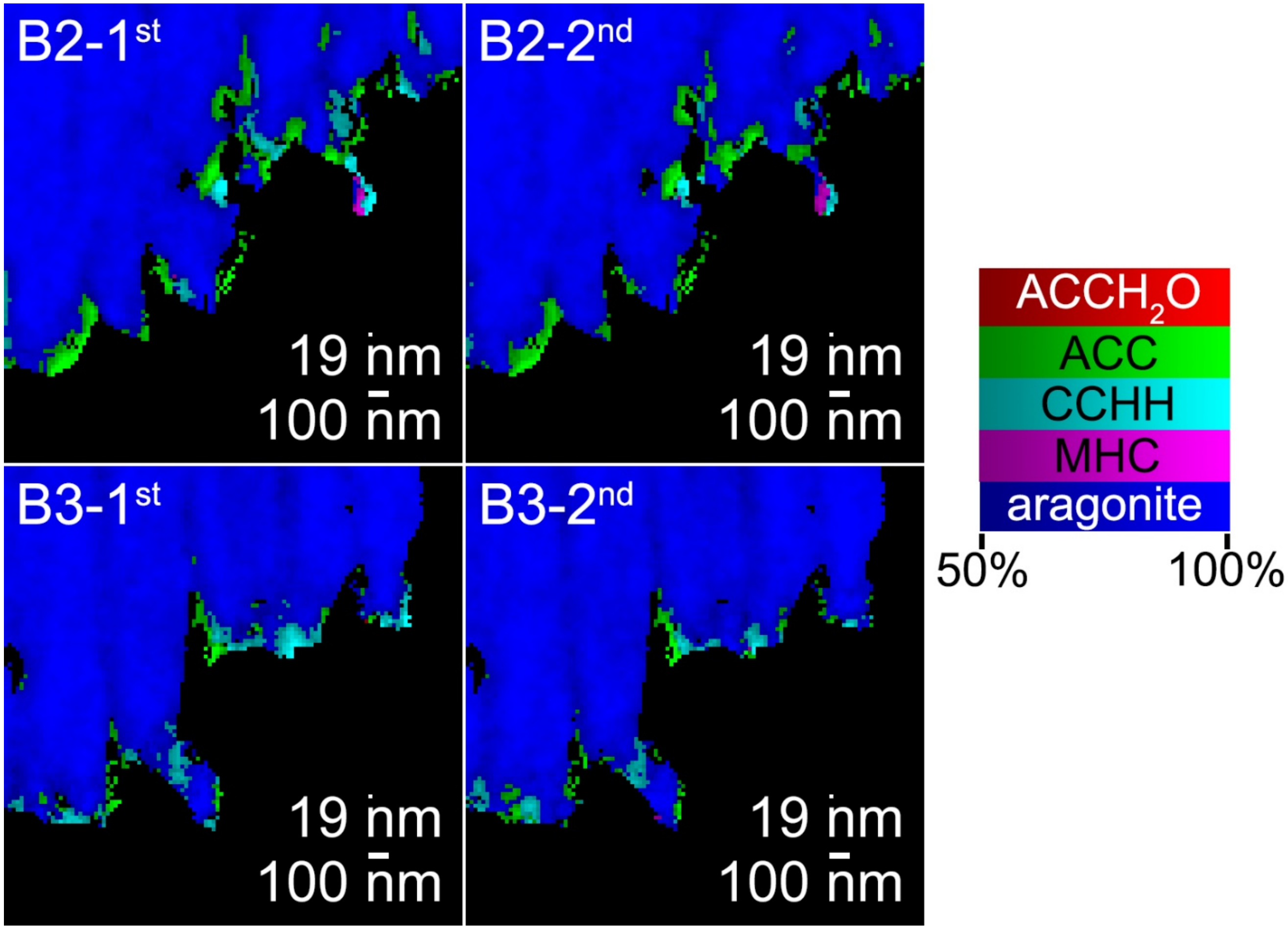

Supplementary Fig. 5. Repeat acquisitions of the same nacre area show phases transforming thermo-dynamically downhill. MMs for the same areas of nacre shown in insets B2 and B3 in Fig. 3, and their repeats. All labels and trends are as described in Supplementary Fig. 4 caption.

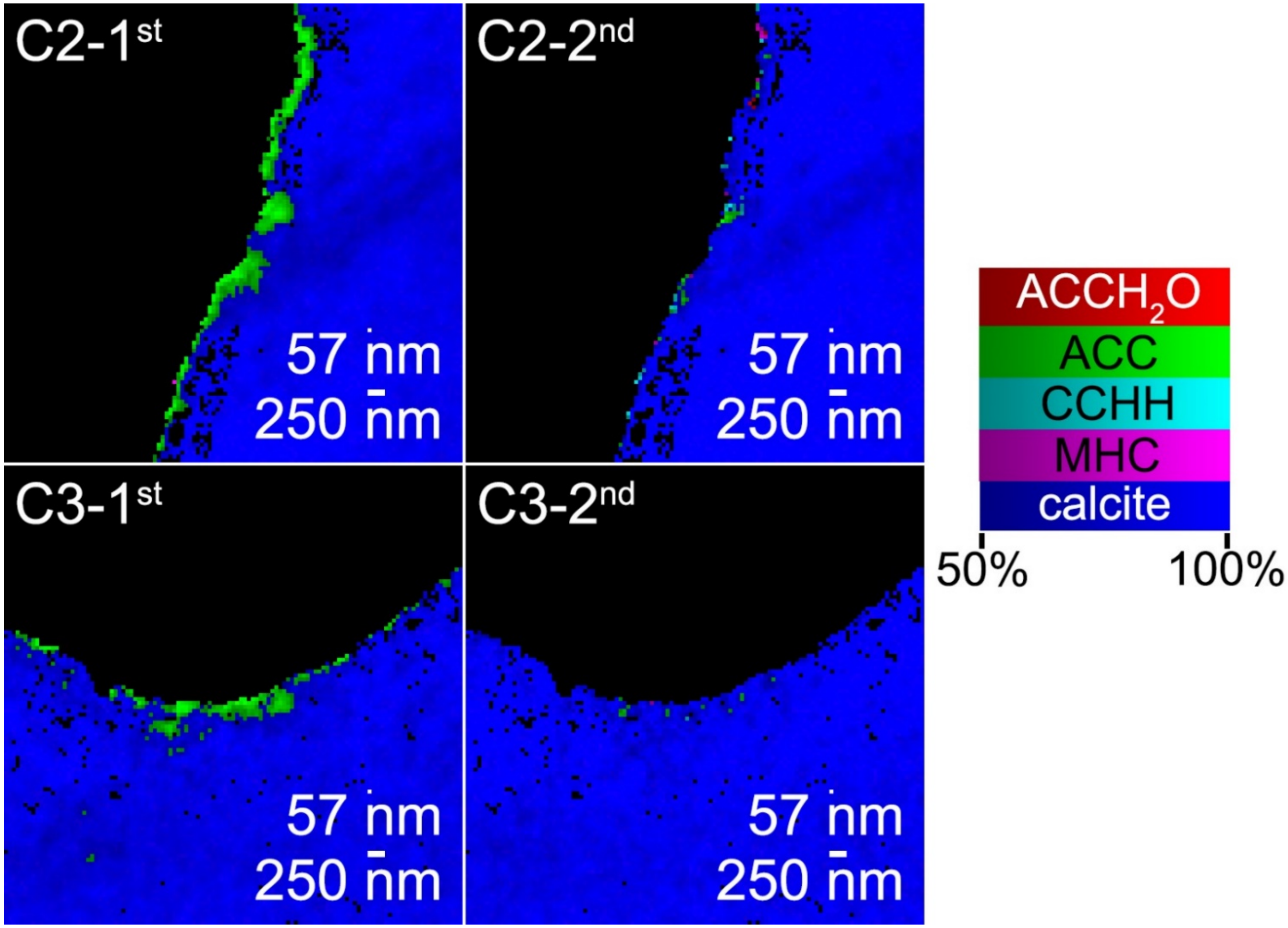

Supplementary Fig. 6. Repeat acquisitions of the same sea urchin spine area show phases transforming thermodynamically downhill. MMs for the same areas of sea urchin spine shown in insets C2 and C3 in Fig. 3 and their repeats. All labels and trends are as described in Supplementary Fig. 4 caption. The only differences here are that the mature mineral is calcite, not aragonite, and CCHH is absent.

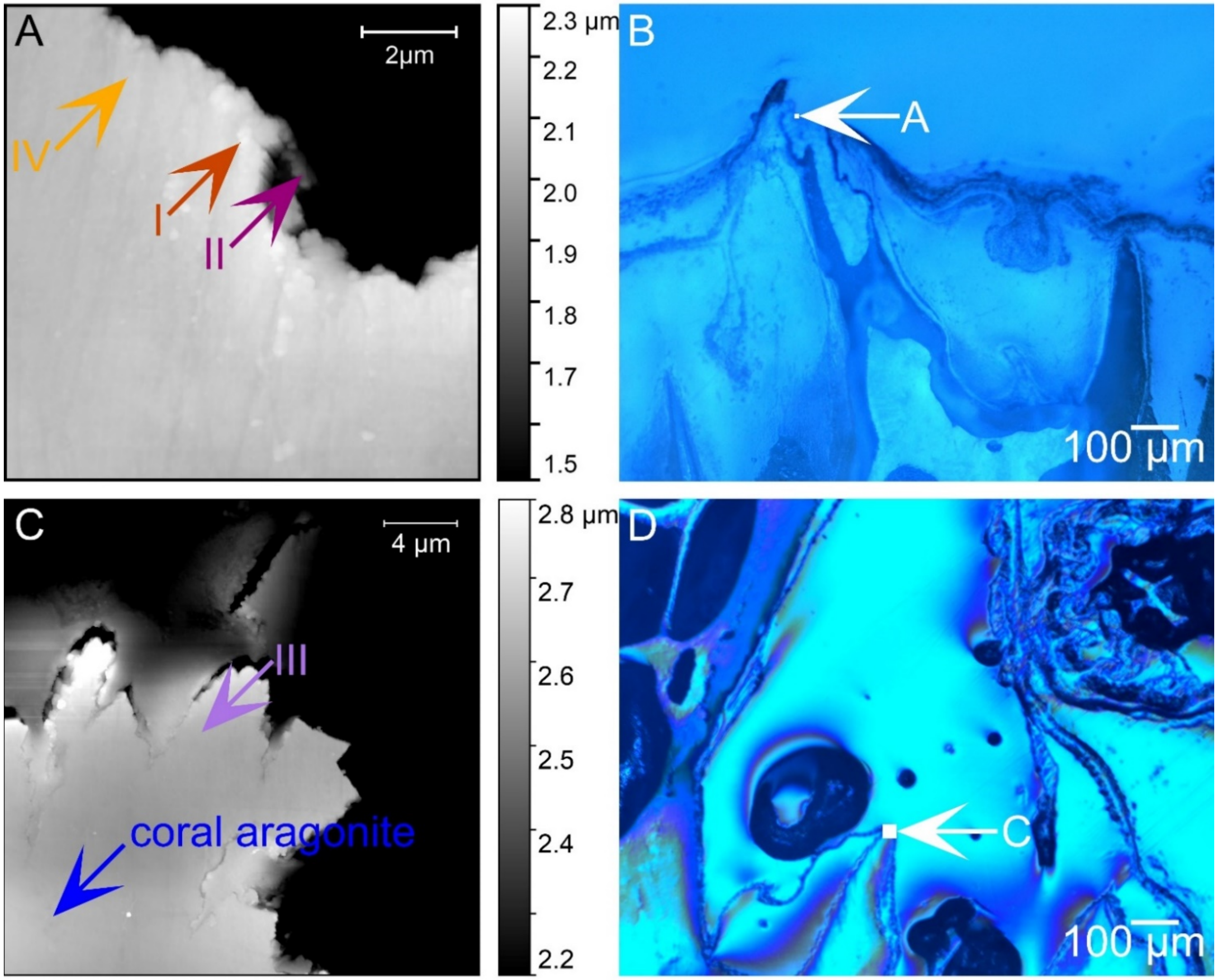

Supplementary Fig. 7. SINS-FTIR data from coral skeletons. A,C. Locations from which the SINS spectra presented in Fig. 4 were extracted from two coral samples. A and C show high magnification AFM topography images with the locations of each spectrum, color coded and labeled as in Fig. 4. Note that the color arrows pointing to the $(20nm)^3$ voxels are substantially larger than the voxels, for clarity. All spectra labeled as "coral surface I-IV" in Fig. 4 are correspondingly labeled here by Roman numerals. These were acquired within 1 μm of the forming surface, whereas the "coral aragonite" spectrum in C was deeper into the mature skeleton. B,D. Polarized light microscopy (PLM) images of the 2 corals samples, in which the areas imaged in A and C are indicated by white boxes, to which the white arrows point. A was at the tip of a nubbin, where two corallites meet, and C was at the tip of a columella.

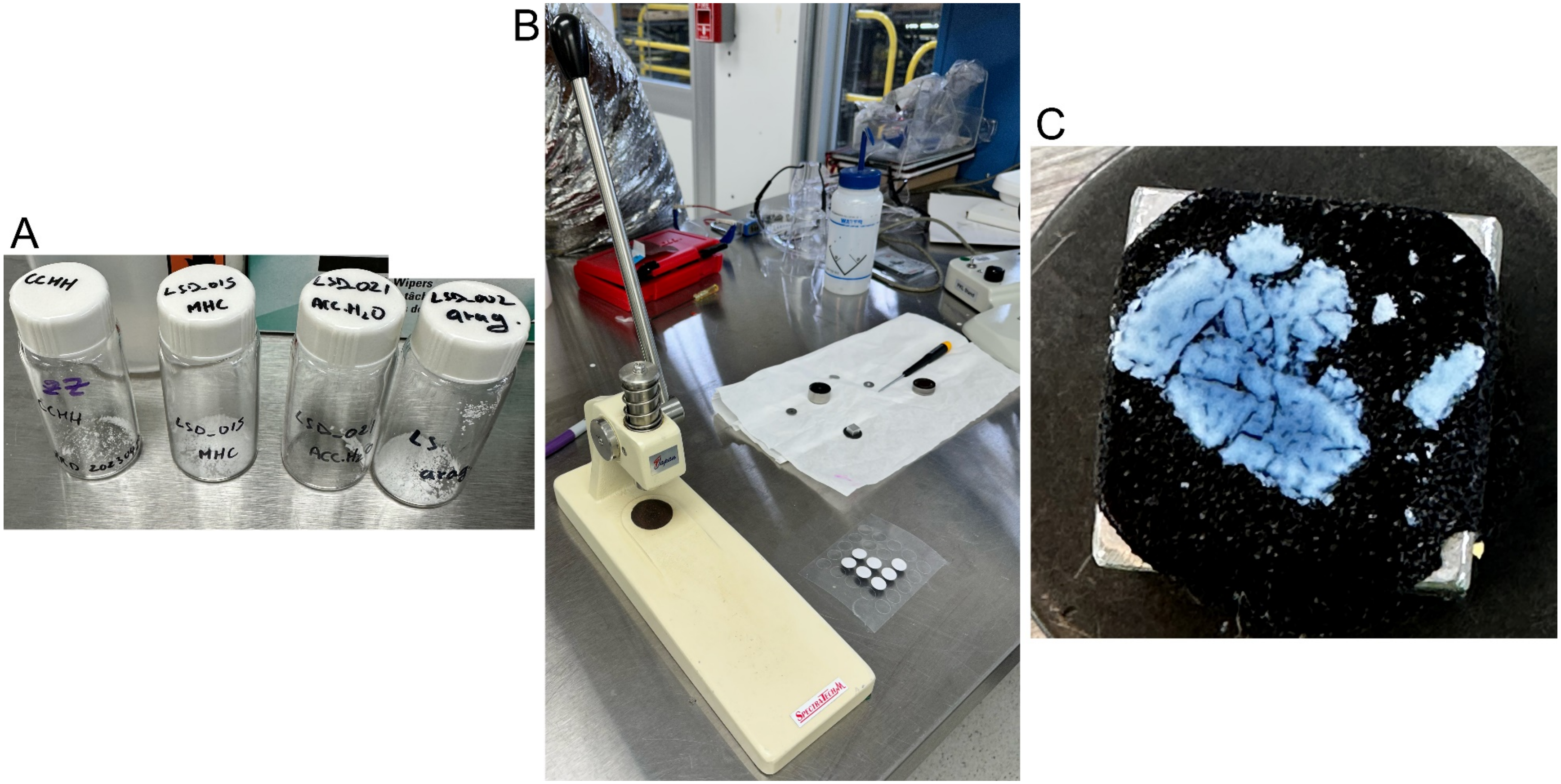

Supplementary Fig. 8. Blue amorphous calcium carbonate. Photographs of A. synthetic mineral powders in glass vials before the experiment, B. the KBr press used to press the powders onto carbon tape disc to obtain a flat, AFM-friendly surface, alongside the aluminum sample pucks and carbon tape discs used. C. A fully prepared synthetic mineral powder after pressing. This is the synthetic $ACCH_2O$, pressed onto a carbon tape disc on an Al puck. No dye or coloring was added to the $ACCH_2O$ in C, it became blue after being pressed. The blue color of $ACCH_2O$ is most evident with a black background. On a white background it appears yellow.

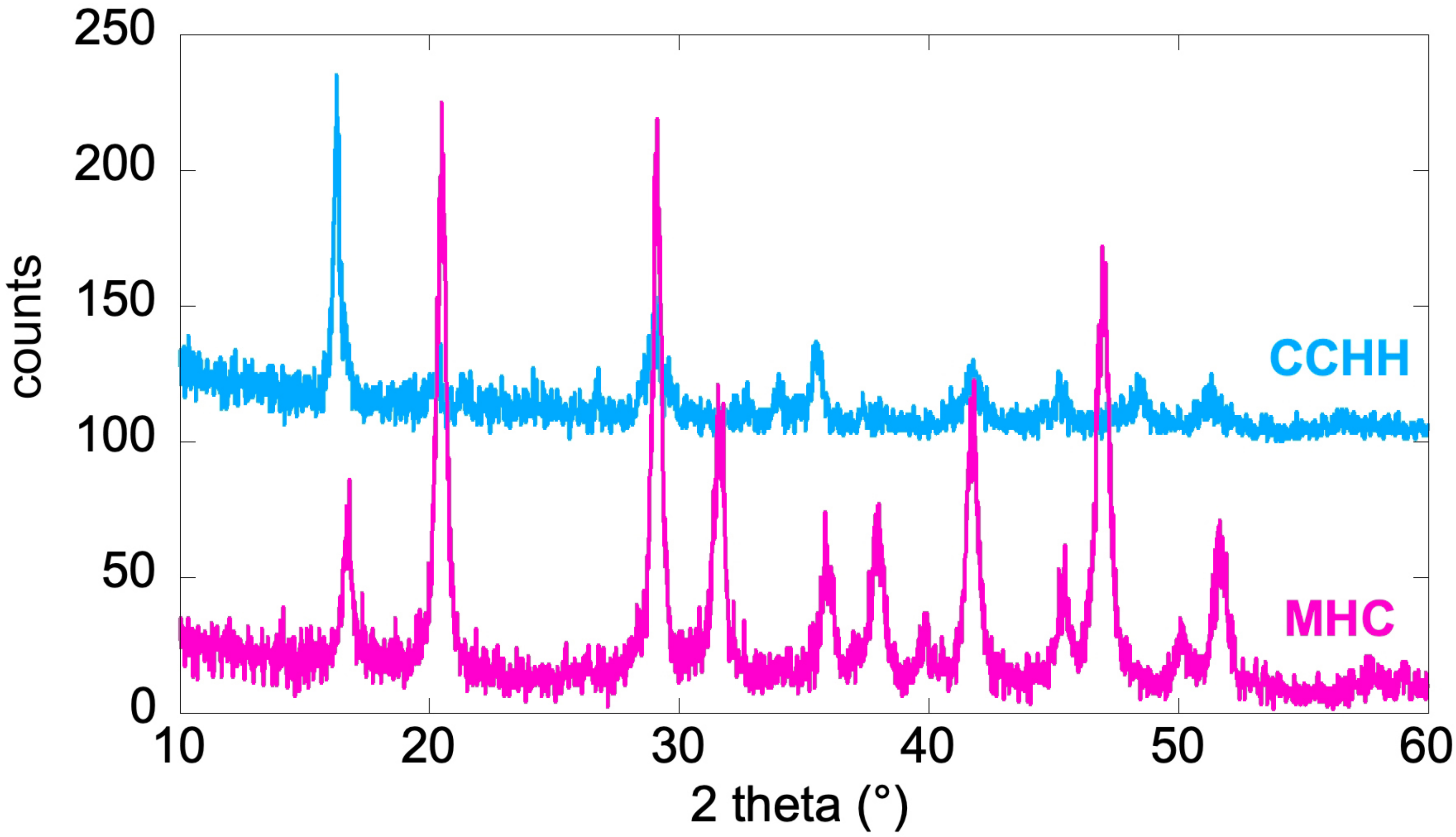

Supplementary Fig. 9. Confirming synthetic metastable mineral phases with diffraction. X-Ray Diffraction (XRD) of synthetic CCHH and MHC analyzed the same day, immediately after the SINS spectra were acquired on these same synthetic powders. These spectra match exactly the ones presented by Zou et al. for the two metastable crystalline phases [2]. The CCHH spectrum is shifted up by 100 counts for clarity.

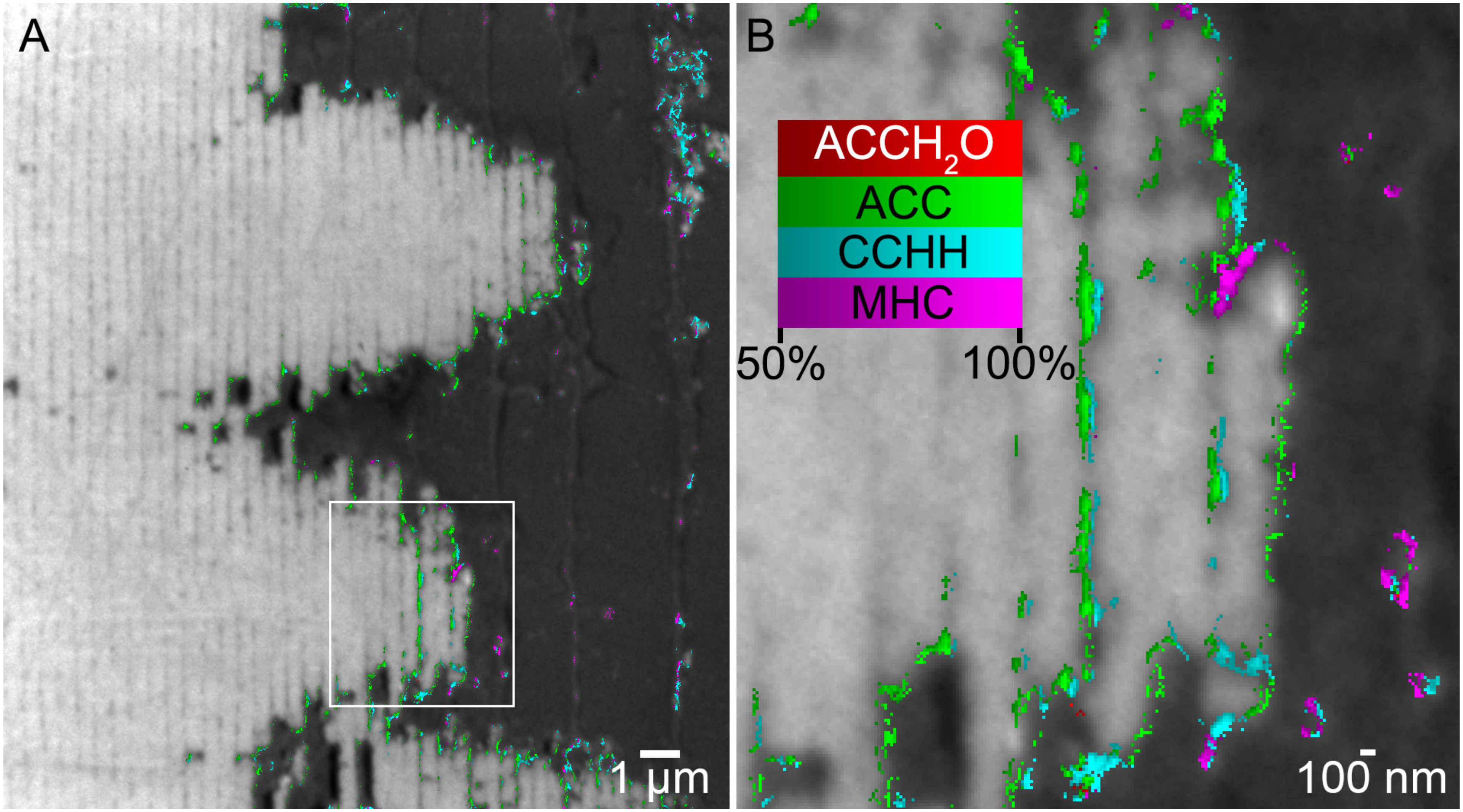

Supplementary Fig. 10. Nacre shows precursors between forming tablets. A. MM of freshly deposited nacre from *Haliotis rufescens*. All component phases are colored as in the color legend here, and all other maps in this work, and blue aragonite is omitted for clarity. Notice the sporadic cyan CCHH and green ACC pixels between forming tablets (center and right side of A), and absence of them in mature nacre (left side of A). B. Magnified region boxed in A, showing ~500 nm thick tablets and ~100 nm-thick layers of mixed ACC and CCHH, which appeared sporadically between forming tablets.

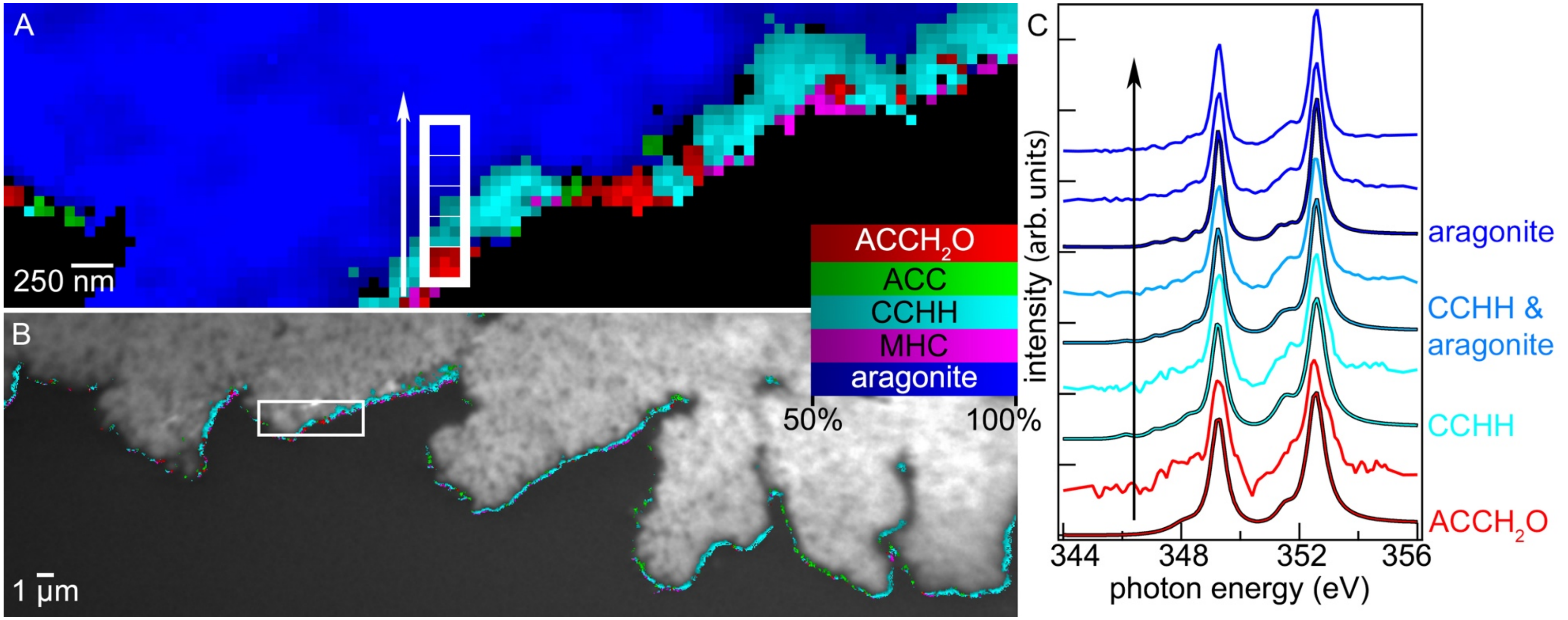

Supplementary Fig. 11. **Spatial sequence of spectra**. A, B. the same data presented in Fig. 2, from a *Stylophora pistillata* coral skeleton surface. The box in B indicates the region magnified in A. The box in A indicates the line along which single-pixel spectra were extracted and displayed in C. This line is 15-pixel long. Five average spectra from each cluster of 9 adjacent pixels along this line were extracted and presented in C, in the direction from outside to inside the skeleton (arrows in A, C). C. Nanoscale carbonate phase x-ray absorption spectra. Five spectra extracted binning 3x3, thus each spectrum is the average of 9 single-pixel spectra (60 nm)^2 x 3 nm deep. The nano-region spectra were interspersed for comparison with the Cni16 spectra (Supplementary Table 1, Supplementary Fig. 1), labeled as $ACCH_2O$, CCHH, and aragonite acquired from synthetic carbonates and color coded as in A and B. The spectra from the pixels in A are immediately above each Cni16 reference spectrum (see Fig. 2C), outlined in black and identically colored. “CCHH & aragonite” is a linear combination of CCHH and aragonite Cni16 spectra, and is colored with light blue, following additive color mixing rules [3]. All spectra are displaced vertically for clarity.

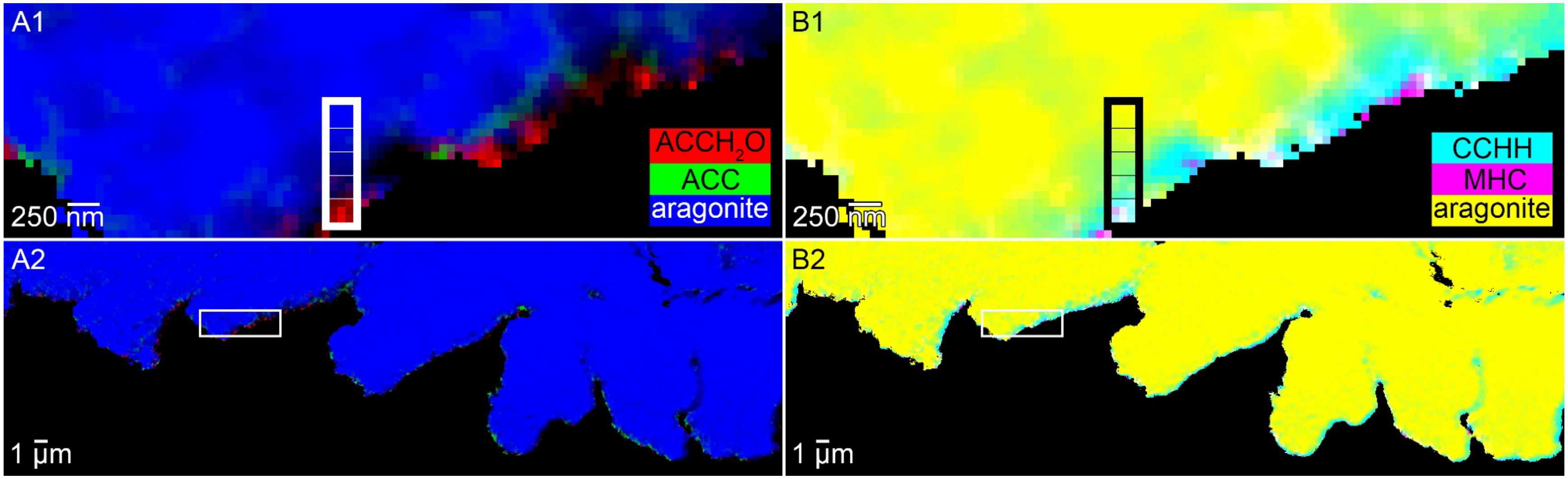

Supplementary Fig. 12. Multiple precursor phases displayed in RGB and CMY instead of MM. The same area shown in Figs. 2, 3, Supplementary Fig. 11 displayed in RGB and CMY color schemes to preserve phase mixing within each map. A. RGB map displaying $ACCH_2O$ and ACC in red and green, respectively, and aragonite in blue. B. CMY map displaying the newly discovered crystalline precursors, CCHH and MHC, in cyan and magenta, respectively, and aragonite in yellow.

Supplementary Files

Supplementary Data 1.xlsx is an Excel single spreadsheet with all Cni16 component spectra used for MM analysis.

Supplementary Data 2.xlsx. is an Excel file with all results for all areas with all χ^2 values. There are 4 spreadsheets:

A. Presents a list of all areas analyzed, and whether each phase appeared in that area. Most of the areas are new. A subset of them were previously published, and this spreadsheet indicates where and when.

B. Presents all 1740 pixels used for χ^2 statistical analysis of CCHH pixels, with all calculations used for Supplementary Table 3.

C. Presents all 194 pixels used for χ^2 statistical analysis of MHC pixels, with all calculations used for Supplementary Table 3.

D. Presents all 295 pixels used for χ^2 statistical analysis of vaterite pixels, with all calculations used for Supplementary Table 3.

E. Presents the results for fitting 30 pixels (6 pixels from each of 5 minerals) with 5 components synthetic (Cni16) vs. biogenic (Cni14), with all calculations used for Supplementary Table 2.

Supplementary Data 3.xlsx is an Excel file with two sheets:

XRD: these are all the XRD results for all synthetic samples used in SINS analysis.

Sample prep: shows the sample preparation recipes also displayed in Supplementary Table 6.

Supplementary References

1. Frazer BH, Gilbert B, Sonderegger BR, De Stasio G. The probing depth of total electron yield in the sub keV range: TEY-XAS and X-PEEM. *Surf Sci* **537**, 161-167 (2003).

2. Zou Z*, et al.* A hydrated crystalline calcium carbonate phase: Calcium carbonate hemihydrate. *Science* **393**, 396-400 (2019).

3. Gilbert PUPA. *Physics in the Arts*, 3rd edn. Academic Press (2021).
