# Supplementary material for: Myriad Mapping of nanoscale minerals reveals calcium carbonate hemihydrate in forming nacre and coral biominerals
Source: Nat Commun. 2024 Feb 28;15:1812. doi: 10.1038/s41467-024-46117-x (PMC10901822; doi:10.1038/s41467-024-46117-x)
Supplement: Supplementary file 3 — Description of Additional Supplementary Files [file 41467_2024_46117_MOESM3_ESM.pdf]

Title: Supplementary Data 1
Description: Excel single spreadsheet with all Cni16 component spectra used for MM analysis.

Title: Supplementary Data 2
Description: Excel file with all results for all areas with all χ 2 values.

There are 4 spreadsheets:

A-all data. Presents a list of all areas analyzed, and whether each phase appeared in that area. Most of the areas are new. A subset of them were previously published, and this spreadsheet indicates where and when. 1.95x108 pixels were analyzed.

B-Table 3, CCHH pixels. Presents all 1740 pixels used for χ 2 statistical analysis of CCHH pixels, with all calculations used for Supplementary Table 3.

C- Table 3, MHC pixels. Presents all 194 pixels used for χ 2 statistical analysis of MHC pixels, with all calculations used for Supplementary Table 3.

D- Table 3, vaterite pixels. Presents all 295 pixels used for χ 2 statistical analysis of vaterite pixels, with all calculations used for Supplementary Table 3.

E- Table 2, all phases. Presents χ 2 statistical analysis of 30 pixels, assigned to all five mineral phases, and all T Test calculations used for used for Supplementary Table 2.

Title: Supplementary Data 3
Description: Excel file with two sheets:

sheet XRD: these are all the XRD results for all synthetic samples used in SINS analysis.

sheet Sample prep: shows the sample preparation recipes also displayed in Supplementary Table 6.
